# A Hypothetical Bottleneck in the Plant Microbiome

**DOI:** 10.3389/fmicb.2018.01645

**Published:** 2018-07-31

**Authors:** George Newcombe, Abby Harding, Mary Ridout, Posy E. Busby

**Affiliations:** ^1^Department of Forest, Rangeland, and Fire Sciences, University of Idaho, Moscow, ID, United States; ^2^Department of Botany and Plant Pathology, Oregon State University, Corvallis, OR, United States

**Keywords:** seeds, maternal transmission, plant microbiome, priority effects, fungi, bacteria

## Abstract

The plant microbiome may be bottlenecked at the level of endophytes of individual seeds. Strong defense of developing seeds is predicted by optimal defense theory, and we have experimentally demonstrated exclusionary interactions among endophytic microbes infecting individual seeds of *Centaurea stoebe*. Having found a single, PDA-culturable microbe per seed or none in an exploratory study with *Centaurea stoebe*, we completed a more extensive survey of an additional 98 plant species representing 39 families. We again found that individual, surface-sterilized seeds of all species hosted only one PDA-culturable bacterial or fungal endophyte per seed, or none. PDA-unculturables were not determined but we expect them to also be bottlenecked in individual seeds, as they too should be governed by exclusionary interactions. If the bottleneck were confirmed with high-throughput sequencing of individual seeds then it would make sense to further investigate the Primary Symbiont Hypothesis (PSH). This includes the prediction that primary symbionts (i.e., the winners of the exclusionary battles among seed endophytes) have strong effects on seedlings depending on symbiont identity.

## Introduction

Seeds (matured ovules or embryonic plants) are the organs of generational and genetic renewal and recombination in plants. What we know of maternal (previously vertical) transmission of microbes in seeds is fragmented as it comes largely from three different ‘schools’: seed pathology, grass endophyte ecology (both of which are of relatively long standing), and more recently, high-throughput sequencing. Grass endophyte ecologists have focused on a relatively specialized system in that it involves host species that comprise just 1% of the plant kingdom. Seed pathologists traditionally focused on incidence of seedborne pathogens in domesticated, crop plants (again, a small fraction of all plants) whereas high-throughput sequencers are focusing on the overall microbiome in a few model plants, and finding abundant diversity in vegetative plant parts. The bottleneck in the plant microbiome that we are proposing here as the basis for the Primary Symbiont Hypothesis (i.e., largely either zero or one isolate of one microbe, bacterium or fungus, per seed), must have been observed by seed pathologists decades ago (e.g., Mundt and Hinkle, 1976). But in that pre-sequencing era that result was not seen in strong contrast to the diversity of the rest of the plant microbiome since that diversity had yet to be revealed. Recent metabarcoding or high-throughput sequencing has revealed that diversity but in bulked seed samples (Klaedtke et al., 2016; Leff et al., 2017; Rezki et al., 2018) rather than in individual seeds. Here, we are emphasizing endophytes of the individual seed, the mechanisms that reduce endophyte diversity at that level, and the consequences of that putative bottleneck in the microbiome of plants.

‘Endophyte’ can be seen as a problematic term since surface-sterilization may fail to completely eliminate epiphytes (Newcombe et al., 2016). This fact makes results reported here conservative in the sense that some of the endophytes we report may actually be epiphytes. We may thus underestimate the severity of the bottleneck. Seed endophytes are more likely to be transmitted maternally and are thought to be less diverse than epiphytes associated with seed coats (Nelson, 2018). Seed epiphytes are fairly diverse (Links et al., 2014) even if they are not as diverse as endophytes in vegetative tissues (Leff et al., 2017). Under ‘Unculturables’ below we will discuss these challenges in targeting endophytes in single seeds.

## Maternally Transmitted Mutualists or Seedborne Pathogens?

Plant biologists have lacked a unified view of maternally transmitted microbes within seeds. In part, this is a product of the divergent interests of plant ecology, on the one hand, and plant pathology on the other. To plant ecologists, grass seeds colonized by systemic *Epichloë* mutualists epitomize the phenomenon of maternal transmission. And to plant pathologists, seeds colonized by fungi (or bacteria, nematodes, viruses, or/spiroplasmas) bring to mind a specific sub-discipline, seed pathology, that has always been focused on the practical elimination of those microbes that are seedborne, crop pathogens. One group of scientists has largely considered maternal transmission of mutualists in a single plant lineage whereas another has searched for maternally transmitted pathogens of relatively few crop species among multiple plant lineages for an applied purpose.

A fragmented understanding of transmission mode has also played a role in impeding synthesis. *Epichloë* mutualists (endophytes of 4,234 species of grass in Pooideae) are maternally transmitted in seed, and it has been assumed that this is because they are also systemic. But, seedborne pathogens, on the other hand, are diverse and some are systemic (e.g., *Ustilago nuda* – Neergaard, 1977) although many are not (e.g., *Pyrenophora tritici-repentis* – Schilder and Bergstrom, 1995). While on the topic of seedborne pathogens we should emphasize that we are not aware of examples of individual seeds bearing more than one pathogen. Given the long history of seed pathology research (Neergaard, 1977) we think it is important to emphasize this.

Transmission of mutualistic *Epichloë* is actually imperfect, or less than 100% (Ravel et al., 1997; Afkhami and Rudgers, 2008; Gundel et al., 2009), and therefore not perfectly systemic. Seedborne pathogens (e.g., *Ustilago nuda*) are also imperfectly transmitted (Newcombe and Thomas, 2000) and similarly not perfectly systemic. The sharp distinction between *Epichloë* mutualists and seedborne pathogens also breaks down at least partially in other ways upon further scrutiny. Meanwhile, zoologists and entomologists are now defining maternal transmission so that it is NOT restricted to a systemic mechanism or transmission mode. Instead, they embrace multiple mechanisms, as outlined below.

## Is Maternal Transmission Universal Among Plants, as It is Among Animals?

Researchers now see maternal transmission of microbes to offspring as universal among animals. “By expanding the definition of maternal transmission to include all internal and external microbial transfers from mother to offspring, we contend that maternal transmission is universal in the animal kingdom and is used to provision offspring with important microbes at birth, rather than leave their acquisition to chance.” (Funkhouser and Bordenstein, 2013). These authors then summarize a remarkable range of diverse transfer mechanisms among mammals, marine invertebrates, marine sponges, vesicomyid clams, and terrestrial invertebrates. Some mechanisms are internal but others, such as ‘egg smearing,’ are external. Insects in particular smear their eggs with symbiont-rich feces. Once hatched offspring then come into contact with shell pieces or even eat them, thus acquiring maternal microbes (Funkhouser and Bordenstein, 2013). In social insects such as bumble bees, the sharing of bacteria with nestmates is essential for defense against gut parasites and is considered to be ‘vertical transmission’ (Koch and Schmid-Hempel, 2011). Even in humans the ‘sterile womb paradigm’ is giving way to a new understanding of frequent, perhaps universal, transmission of microbes to infants before birth. Some maternally transmitted microbes can be essential in tuning the immune system (Hooper et al., 2012) but others cause diseases. For example, intrauterine infections with various, microbial pathogens (e.g., *Listeria monocytogenes*) cause miscarriages or preterm labor and birth associated with infant mortality. Congenital diseases caused by microbial pathogens include the TORCH group: taxoplasmosis, other (syphilis, varicella zoster, parvovirus), rubella, cytomegalovirus and herpes simplex. Infants can be born infected with, and already suffering from, the Zika virus (Brasil et al., 2016) or with HIV that places these ‘vertically infected’ children at much greater risk of mortality than uninfected children (Newell et al., 2004). So, in the animal world generally both pathogens and mutualists are maternally transmitted by transfer mechanisms that may be as varied as animal species themselves are. Transmission rates presumably vary among mechanisms but perfect (100%) transmission appears to be rare.

Maternal transmission defined in this way could well be universal among plants but the question has not drawn sufficient attention to individual seeds. Many plant scientists may even be unfamiliar with the term, or its older equivalent ‘vertical transmission.’ When offered, the definition is typically mechanism-focused (Saikkonen et al., 2004): “Vertical transmission: transmission of the systemic fungus from plant to offspring via host seeds.” Another more detailed but similar definition is this recent one: “The associations between grasses and ‘type 1’ endophytic fungi of the Clavicipitaceae family have been well documented, as the latter colonize the host systemically, and are vertically transmitted in a classic example of mutualism.” (González-Teuber, 2016). This has been taken to mean a systemic transmission mechanism that is perfect (i.e., 100% transmission to seeds), in line with mutualistic benefits that are expressed in all environmental circumstances. But, both aspects of that perspective are actually problematic. *Epichloë* endophytes may often be defensive mutualists, but in some species and populations, and in some environmental circumstances, they tend not to be (Faeth, 2002; Schardl et al., 2004). Transmission of endophytic *Epichloë* is also typically less than 100%, in spite of the systemic mechanism (Ravel et al., 1997; Afkhami and Rudgers, 2008; Gundel et al., 2009). Moreover, imperfect transmission is by no means restricted to *Epichloë*. Vertical transmission of mutualistic *Stagonospora* species in *Phragmites*, common reed, is also imperfect (i.e., 13 isolates from 598 seeds) even though these fungi are found in all plant organs, a pattern suggestive of systemicity (Ernst et al., 2003). A third example of systemicity was recently reported (Soares et al., 2016) that also involves very imperfect transmission: *Bacillus amyloliquefaciens* in English ivy (*Hedera helix*), in which the bacterium systemically colonized leaves, petioles, and seeds. The bacterium exerted powerful hormonal growth effects and protected its plant host from disease caused by *Alternaria tenuissima*, yet the frequency of its isolation from *Hedera helix* seeds was very low (i.e., very imperfect): between 1 and 3% depending on isolation medium.

A more inclusive definition of maternal transmission in plants appears to be on the horizon for plant biologists. It could include what was recently called ‘pseudo-vertical transmission’ because the microbes in question were effectively transmitted to the next generation in fruit tissues (Séne et al., 2018). Still, a more inclusive definition of maternal transmission is tangential here to the question of a bottlenecked seed microbiome. In fact, some might assume that with both systemic and non-systemic microbes in seed, a given seed microbiome might be at least as diverse as its vegetative counterpart. But is it? And what reasons might there be for a microbial transmission bottleneck in seeds?

## On What Grounds Can a Maternal Transmission Bottleneck be Hypothesized for Endophytes in Plants?

In a paper published 42 years ago, Mundt and Hinkle (1976) presented evidence for a bacterial bottleneck in seeds by culturing from surface-sterilized seeds and ovules of 27 plant species. “Bacteria were obtained from 30% of the ovules, 15% of the seeds of herbaceous plants, 16% of the seeds of woody plants, 5.4% of the overwintered non-cereal seeds, and 13.5% of overwintered cereal seeds… In no instance did every ovule or seed of a plant species contain bacteria.” This evidence for a bacterial bottleneck in seeds was obtained from culture in the water of syneresis of a nutrient medium low in agar content. Fungi were not included, and other bacteria might have been isolated with a different protocol. Still, this study provided something of an early basis for the hypothesis of a microbial bottleneck in seeds.

Additional indications of a restricted seed microbiome derive from endophyte studies showing that dominant, culturable microbes in leaves could be absent or rare in seeds, and/or that microbes were less common in seeds than vegetative plant parts. For example, *Xylaria* species can dominate tropical leaf microbiomes (e.g., those of *Casuarina equisetifolia* and *Manilkara bidentata* – Bayman et al., 1998) but are typically absent from seeds. Similarly, *Discula quercina* is the dominant endophyte of *Quercus garryana* in the maritime Pacific Northwest but it is rare in seed (Wilson and Carroll, 1994). In a study of white pine endophytes, only 16 isolates were obtained from 800 seeds of *Pinus monticola*, with one isolate per seed (Ganley and Newcombe, 2006). This finding was in contrast to a yield of 2003 fungal isolates from 750 leaves (needles) of the same species. In other words, the seed isolation frequency of endophytes was roughly 125 times greater than the needle equivalent. Moreover, the dominant members of the foliar microbiome of *Pinus monticola* were not represented at all among the 16 seed isolates. In particular, *Lophodermium* species were absent from seed even though they dominate white pine needles (Ganley and Newcombe, 2006). In yet another endophyte study, 15 distinct isolates were obtained from seeds of *Atriplex canescens* (Lucero et al., 2011); presumably, more diversity would be obtained from the vegetative microbiome of that species. Directly sequenced leaves of *Populus balsamifera* (*N* = 153) for example, yielded over 2000 fungal OTUs (Bálint et al., 2015). In leaves and sapwood of *Hevea brasiliensis* 225 samples yielded 175 isolates that resolved to 58 distinct, sequence-based OTUs (Gazis and Chaverri, 2010).

Further suggestions of a bottleneck are found in studies of bacterial endophytes. Endophytic bacteria are in seed in many species and hybrids of *Eucalyptus* although “seed densities were low compared with plant vegetative densities” (Ferreira et al., 2008). In a second study of endophytic bacteria (Compant et al., 2011), 1.44 ± 1.44 log10 CFU g-1 were isolated from seeds compared to 7.73 ± 0.4 log10 CFU g-1 as rhizobacteria, 5.92 ± 1.43 log10 CFU g-1 as root endophytes and 3.69 ± 0.1 log10 CFU g-1 as endophytes in flowering stalks. In a more recent study focused on just one bacterial species (Soares et al., 2016) that we have already briefly discussed, ‘systemic’ *Bacillus amyloliquefaciens* was isolated from seed at 1.2% and 2.7% on 10% TSA and 1% YES media, respectively, whereas the isolation frequencies of this bacterium from mature leaves were 62.5 and 93%.

## A Basis in Theory?

There is a theoretical basis for a seed microbiome bottleneck: optimal defense (Stamp, 2003). It predicts stronger defense of seeds than of vegetative plant parts. In keeping with this, seeds are predicted to contain the highest levels of defense compounds within the plant (Zangerl and Bazzaz, 1992; Zangerl and Berenbaum, 1997). In particular, for monocarpic plants (i.e., those that produce seed only once per generation) selection for seed fitness is also expected. In *Arabidopsis thaliana* (Nour-Eldin et al., 2012), methionine- and tryptophan-derived glucosinolates, the major defense compounds, accumulate throughout the plant prior to flowering. But, with flowering and seed development, concentrations in leaves decline as levels in seeds increase. As seeds are unable to synthesize glucosinolates, import of these defense compounds from other tissues must occur, and this has been confirmed experimentally. Moreover, these strong seed defenses appear to have consequences for the seed microbiome of *Arabidopsis*; molecular methods have been used to confirm the endophytic sterility of a sample of seeds of *Arabidopsis thaliana* (Lundberg et al., 2012). Findings similar to the *Arabidopsis* study (i.e., a bottlenecked seed microbiome) have been reported for tobacco (Ohnmeiss and Baldwin, 2000). These findings of seed microbiomes of reduced diversity again stand in sharp contrast to vegetative microbiomes that are staggeringly diverse for a given species (e.g., Arnold, 2007).

Still, seed microbiomes of a given plant species do exhibit some diversity. For example, in *Centaurea* seed we found 92 sequence-based OTUs among 2291 fungal isolates from 8763 seeds (Shipunov et al., 2008). These 8763 field seeds of this species were collected both from sites in North America and in Eurasia; 26% of seeds yielded one, PDA-culturable isolate per seed and 74% yielded none. We did not also estimate the number of OTUs in seed-sized sample units of the vegetative microbiome of *Centaurea stoebe.* But, we suspect that the number would have been much higher than 92. For one thing, in *Centaurea* greenhouse studies we always were able to isolate ‘greenhouse microbes,’ lacking host specificity, from leaves, roots and stems, even when the plants were started from endophyte-free seed. In contrast, endophyte-free seed (i.e., free of culturable endophytes) of *Centaurea* were always obtained in the greenhouse in the absence of inoculation with *Centaurea* seed endophytes (Aschehoug et al., 2012). In other words, the greenhouse fungi that infected vegetative parts of the plant were not able to infect developing seeds even when we provided optimal conditions for infection.

However, even if the *Centaurea* seed microbiome is less diverse than its vegetative counterpart, there were still 92 OTUs overall. If all 92 OTUs were present in an individual seed, the latter would hardly be bottlenecked. But again, in individual seeds we found either one culturable isolate per seed or none in virtually all cases (Shipunov et al., 2008; Raghavendra et al., 2013).

## What is the Cause of this Bottleneck at the Level of the Individual Seed?

Many endophytes isolated from leaves and roots cannot infect developing seeds. We even found that isolates from *Centaurea* seeds would frequently fail to infect developing seeds when inoculated into flowers (Raghavendra et al., 2013). In a similar manner, Darrasse et al. (2018) could not obtain any infection of seeds of *Phaseolus vulgaris* with non-pathogenic strains of *Xanthomonas citri* pv. *fuscans* even though pathogenic strains could infect. These outcomes can be attributed to host resistance.

But, a second mechanism contributes greatly to the bottleneck at the individual seed level: inhibitory interactions among seed endophytes (Raghavendra et al., 2013). These interactions were so strong as to be completely exclusionary. This finding was based on deliberate inoculation of flowers in the greenhouse with pairs of seed isolates. We provided optimal conditions for infection of each pair of isolates. Yet, invariably only one isolate was successful, and across seeds, flowerheads and *Centaurea* genotypes, it was always the same one. For example, if we mixed a *Cladosporium* seed isolate with a *Botrytis* and inoculated flowers (i.e., developing seeds) all isolates in the resulting mature seeds, one isolate per seed, were *Cladosporium*. In not a single case did *Botrytis* exclude *Cladosporium.* Yet, if we mixed that same isolate of *Cladosporium* with a *Fusarium*, all isolates in the mature seeds were that *Fusarium*, again one per seed; *Cladosporium* was always excluded. *Fusarium* also always excluded *Botrytis*. Even though our results demonstrated a transitive system, further research might well reveal a non-transitive system overall. With a PCR-based method, we showed that the interactions were exclusionary and not merely suppressive (Raghavendra et al., 2013). This exploratory work suggested, in sum, that it is exclusionary interactions among microbes that limit infection to one culturable endophyte per seed. We had hypothesized plant genotype but it did not contribute to these exclusions and thus did not contribute to the bottleneck (Raghavendra et al., 2013). Subsequent inoculations of various plant species in the greenhouse and field have also failed to break the bottleneck of individual seeds by increasing the number of endophytic isolates per seed (Newcombe et al., unpublished). Other examples of microbe–microbe, exclusionary interactions are known (e.g., that of *Verticillium longisporum* and *Paenibacillus polymyxa* – Rybakova et al., 2017).

## How General is this Binary, ‘Zero/One’ Pattern?

We report here an overview of a survey of PDA-culturable microbes at the individual seed level in samples (*N* = 70) from 98 plant species from 39 families (**Table 1**), following agitation (1 min in a 0.83% solution of Tween^®^ 20 (Sigma-Aldrich), surface-sterilization (1 min in 70% ethanol), and rinsing (1-min in sterile distilled water). Roughly half of the 98 species (i.e., 47 spp.) were represented by seeds collected in nature or in the UI Arboretum; the other half (i.e., 51 spp.) were purchased from commercial sources. Seeds were then plated onto 4% potato dextrose agar. Our use of PDA in our 98-species survey was deliberate as it allows for the culture of a wide range of bacteria and fungi (Atlas, 1997).

**Table 1:** Proportion of seeds either without (i.e., ‘zero’) or with one or two PDA-culturable endophytes per surface-sterilized seed (70 seeds per species for 98 plant species from 39 families).

Plant Family	# of species tested^1^	Zero	One	Two
Actinidiaceae	1	1	0	0
Adoxaceae	1	1	0	0
Amaranthaceae	1	0.79	0.20	0.01
Amaryllidaceae	1	0.63	0.36	0.01
Apiaceae	6	0.51	0.45	0.04
Asteraceae	12	0.57	0.37	0.06
Boraginaceae	1	0.13	0.79	0.09
Brassicaceae	6	0.82	0.18	0.01
Caprifoliaceae	3	0.88	0.10	0.02
Caricaceae	1	0.96	0.04	0
Chenopodiaceae	2	0.87	0.13	0
Convolvulaceae	1	0.06	0.89	0.06
Cucurbitaceae	3	0.30	0.67	0.03
Cupressaceae	1	0.96	0.04	0
Cyperaceae	1	0.94	0.06	0
Elaeagnaceae	1	0.99	0.01	0
Ephedraceae	1	0.77	0.13	0.10
Ericaceae	2	0.91	0.09	0
Fabaceae	9	0.76	0.22	0.02
Gentianaceae	1	0.99	0.01	0
Juglandaceae	1	0.85	0.13	0.02
Lamiaceae	3	0.70	0.30	0
Liliaceae	1	0.79	0.20	0.01
Linaceae	1	0.99	0.01	0
Nyctaginaceae	1	0	0.73	0.27
Onagraceae	1	0.99	0.01	0
Orchidaceae	1	1	0	0
Papaveraceae	4	0.45	0.40	0.15
Pedaliaceae	1	1	0	0
Pinaceae	2	0.59	0.39	0.02
Poaceae	11	0.35	0.56	0.10
Polygonaceae	1	0.40	0.59	0.01
Rosaceae	9	0.89	0.11	0.01
Rubiaceae	1	0	1	0
Rutaceae	1	0.99	0.01	0
Salicaceae	1	1	0	0
Sapindaceae	1	0.31	0.57	0.11
Solanaceae	1	0.19	0.81	0
Tiliaceae	1	1	0	0
Totals	98	0.70	0.27	0.03


*Approximately 70% were endophyte-free zeros; 27% produced one isolate, the primary symbiont. Seeds with two isolates per seed were only 3% overall.*

*^1^The 98 species are listed in the alphabetical order of their respective families: Actinidia deliciosa, Sambucus cerulea, Amaranthus cruentus, Allium cepa, Anethum graveolens, Carum carvi, Coriandrum sativum, Daucus carota, Foeniculum vulgare, Perideridia gairdneri, Balsamorhiza sagittata, Tagetes patula, Centaurea stoebe, Cirsium arvense, Helianthus annuus, Hieracium caespitosum, Lactuca sativa, Lactuca serriola, Silybum marianum, Taraxacum officinale, Tragopogon dubius, Zinnia elegans, Cynoglossum amabile, Boechera stricta, Brassica rapa, Lunaria annua, Raphanus sativus, Sinapis alba, Thlaspi arvense, Symphoricarpos albus, Valerianella locusta, Carica papaya, Lonicera syringantha, Chenopodium quinoa, Spinacia oleracea, Ipomoea quamoclit, Cucumis melo, Cucumis sativus, Cucurbita pepo, Cupressus bakeri, Scirpus tabernaemontani, Elaeagnus multiflora, Ephedra minuta, Arctostaphylos uva-ursi, Pterospora andromedea, Caragana arborescens, Lens culinaris, Lupinus sericeus, Medicago sativa, Phaseolus vulgaris, Trifolium pretense, Trigonella foenum-graecum, Vigna angularis, Vigna radiata, Frasera speciosa, Juglans nigra, Ocimum basilicum, Origanum vulgare, Salvia hispanica, Calochortus nuttallii, Linum lewisii, Mirabilis jalapa, Epilobium ciliatum, Vanilla planifolia, Eschscholzia californica, Papaver nudicaule, Papaver orientale, Papaver somniferum, Sesamum indicum, Larix occidentalis, Pinus ponderosa, Bromus tectorum, Hordeum vulgare, Oryza sativa, Pennisetum glaucum, Poa secunda, Pseudoroegneria spicata, Secale cereale, Triticum aestivum, Ventenata dubia, Zea mays, Fagopyrum esculentum, Amelanchier alnifolia, Crataegus douglasii, Fragaria x ananassa, Physocarpus malvaceus, Potentilla glandulosa, Purshia tridentata, Rosa gymnocarpa, Rubus macrophyllus, Rubus parviflorus, Coffea arabica, Citrus x limon, Populus trichocarpa, Acer glabrum, Acer platanoides, Capsicum annuum, and Tilia americana.*

Overall, 70% of all surface-sterilized seeds were ‘zeros’ in that no isolates were obtained; 27% were ‘ones,’ with a single, primary symbiont of varying identity. Two or more culturable endophytes were hosted by only 3% of the seeds overall. Seed weight and germination did not affect this binary pattern. In four, exceptional species more than 20% of seeds yielded two or more isolates: *Eschscholzia californica* (37%), *Mirabilis jalapa* (27%), *Papaver orientale* (23%) and *Secale cereale* (60%). Thus, in only the one, latter species (i.e., domesticated rye) were seeds with more than one isolate in the majority.

Nine of the 98 species yielded no isolates at all (*N* = 70 seeds per species): *Actinidia deliciosa, Cirsium arvense, Populus trichocarpa, Sambucus cerulea, Sesamum indicum, Tilia americana, Trifolium pretense, Valerianella locusta*, and *Vanilla planifolia*. With larger sample sizes, media in addition to PDA, and PCR-based attempts to detect ‘unculturables,’ we would presumably find some isolates even in these species. We have, for example, obtained *Zea mays* seed isolates of *Aspergillus* with glycerol agar that are never obtained on PDA. Conversely, a species of *Pichia* yeast is commonly isolated from *Z. mays* seed on PDA that is not obtained on glycerol agar. There might also be inter-annual variation; with *Populus trichocarpa* a 2018 study of 1,380 seeds dissected aseptically (rather than surface-sterilized) from capsules of 23 female trees did yield bacterial and fungal isolates on PDA, and all were one isolate per seed (Ridout et al., unpublished).

We will report elsewhere on the identities of the isolates from seeds of the 98 species. But, in general with PDA we found more bacteria than fungi: 1683 versus 1156 isolates, respectively. There were also trends among plant families. Seeds of the three species of Cucurbitaceae, for example, were dominated by bacterial isolates: 149 versus three fungal isolates. In contrast, the nine species sampled from the Rosaceae yielded only one bacterial isolate for every three fungal isolates.

## Unculturables?

It is surprising that pursuit of the core microbiome has not yet led to use of widely adopted, culture-independent, high-throughput sequencing methods at the level of individual seeds to confirm or refute a putative trans-generational bottleneck in the transmission of endophytes. In looking for publications we found Truyens et al. (2015) who cited 44 papers characterizing endophytes of seeds. Of these 44 only one (i.e., Mundt and Hinkle, 1976) recorded isolation on a per-seed basis, and that was in the era before high-throughput sequencing. In a recent summary, Nelson (2018) stated that “despite the rapidly increasing emphasis on microbiome studies, seeds are rarely mentioned”. In a high-throughput sequencing study that included seeds (Leff et al., 2017), those of *Helianthus annuus* were “prepared by soaking the seeds in DNA-free water for 24 h, briefly submerging in 95% ethanol, and rinsing with water” so as to “soften seeds and remove superficial microbial cells, but microbial cells integrated in the seed coat were deliberately retained because they could influence the adult plant’s microbial community.” So, the more diverse seed epiphytes were included with the less diverse endophytes (Nelson, 2018). Still, sunflower “fungal and bacterial diversity in seeds was lower than in root and rhizosphere communities.” The possibility of a maternal transmission bottleneck was not discussed. Hopefully, this paper will stimulate attempts to confirm or refute a putative trans-generational bottleneck with high-throughput sequencing methods at the level of individual seeds.

**FIGURE 1:**
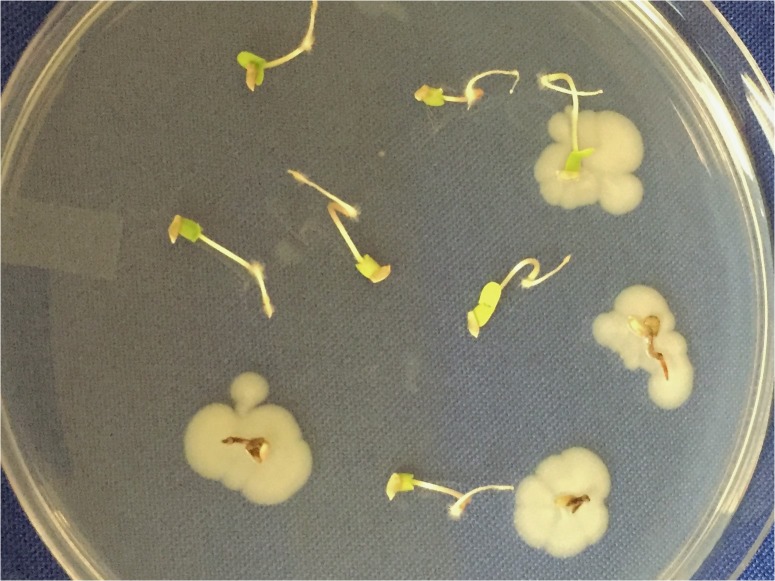
Example of the ‘natural experiment’. Ten seeds of *Populus trichocarpa* from a mature capsule (from a female tree along the Clearwater River in Idaho) were opened in a laminar flow cabinet and then germinated on PDA to allow primary symbionts to emerge. In this case, six seedlings were free of culturable symbionts whereas four hosted *Pseudomonas syringae*, that was lethal to three of the four. In general, seedlings hosting primary symbionts can then be transplanted aseptically for inoculations with secondary symbionts, (e.g. DSE fungi, as discussed in the text) and contrasted with those free of culturable symbionts.

High-throughput sequencing to detect ‘unculturable’ endophytes in individual seeds will have to deal with the problem of epiphytes and surface contaminants, alluded to earlier. Two methods are being explored. One involves sequencing seed-washing fluid to determine the epiphytes and surface contaminants. The other involves dissection of fruits under laminar flow and aseptic removal of seeds (e.g., **Figure 1**) that were thus never exposed to airborne contaminants.

With high-throughput sequencing of individual seeds we predict a slightly higher overall rate of maternal transmission than what we found with PDA isolation of seeds of our 98 species. However, we still maintain that the bottleneck at the level of individual seeds would remain because exclusion should operate as effectively among ‘unculturables’ as it does among culturables. This corollary will be difficult to test via manipulative experiment until media are found to culture those same PDA-unculturables.

It is also possible that even accounting for ‘unculturables,’ seeds without symbionts (i.e., ‘zero’ seeds) are common in some plants. Partida-Martinez and Heil (2011) have speculated on the existence of ‘microbe-free plants’ as young germinants and seedlings. Even the world’s second-largest seeds (i.e., those of *Cocos nucifera* – second in size only to seeds of *Lodoicea maldivica*) are said to be sterile, at least until their physical integrity is compromised (Maciel et al., 1992). Similarly, the endophytic sterility of fresh cacao seeds has made possible the groundbreaking studies of the roles of endophytes in plant defense (Arnold et al., 2003; Christian et al., 2017) since ‘zero’ seeds and/or seedlings can be inoculated without having to worry about the interference of maternally transmitted microbes.

## The Primary Symbiont Hypothesis (PSH)

Plants host primary, or sole, or at least dominant, endophytic symbionts in individual seeds (the first part of the PSH). The presence/absence and identity of the primary symbiont in each seed affects survival of the host in the vulnerable stages of seed dispersal, germination, emergence and young growth (i.e., the second part of the PSH). If culture-independent, high-throughput sequencing at the level of individual seeds reveal several OTUs per seed there still might be a ‘primary symbiont’ in the form of a dominant one with effects on fitness of seedlings.

## Evidence in Support of the Second Part of the PSH

We have discussed the evidence for the first part of the PSH. For the second part, evidence for the importance of seed symbionts in young germinants and seedlings was published recently (Johnston-Monje et al., 2016). There are dynamic changes in the seed microbiome as seeds germinate and emerge (Barret et al., 2015). Primary seed symbionts had significant, fitness-altering effects on plant-plant competition when inoculated into *Centaurea stoebe* seedlings that were grown from endophyte-free seed (Aschehoug et al., 2012, 2014). Modification of disease severity may be a general way in which primary symbionts contribute to seedling fitness (Busby et al., 2016a,b; Ridout and Newcombe, 2016). On the other hand, some primary symbionts are, of course, lethal pathogens, so we are not suggesting fixed mutualism. In fact, it is the broadness of the range from strong mutualism to strong parasitism that should make identity of the primary symbiont so important. Determination of the full range of indirect interactions will take time. But in employing what we call the ‘natural experiment’ (**Figure 1**) we have already found that primary symbionts strongly affect recruitment of dark-septate endophytes (DSE) in the *Phialocephala fortinii* s.l. – *Acephala applanata* species complex (Ridout, unpublished). DSE colonization can in turn have effects on fitness via various mechanisms (Jumpponen and Trappe, 1998; Stroheker et al., 2018).

## More Attention Should be Given to Individual Seeds

Neglect of primary symbionts in individual seeds has no doubt contributed to unexplained variation in past inoculation studies. For example, primary symbionts might even underlie the context-dependency that plagues many functional studies based on inoculation with endophytes (Chamberlain et al., 2014). Primary symbionts might also have been confounded with host genotype as factors structuring microbiomes. Via the natural experiment outlined in **Figure 1** we have shown that primary symbionts strongly influence early assembly of individual plant microbiomes (Ridout, unpublished). Another recent review also makes this point that further microbiome assembly might hinge upon what is maternally transmitted (Shade et al., 2017).

## Concluding Remarks

Wild plants experience high mortality early followed by an increase in life expectancy with age. Undomesticated plants are thus among organisms with Type III survivorship curves (Demetrius, 1978) because large progenies are winnowed down to just a few survivors. In contrast, the Type I (e.g., human) survivorship curve is characterized by low probability of mortality in youth and even in middle age. The PSH is proposed to be true for plants generally but it is worth noting that agricultural and horticultural plants receive human care that greatly reduces mortality among seedlings. This care has always distorted the Type III curve of domesticates with cumulative but still unexamined consequences for maternal transmission of microbes.

Strong defense of seeds can affect not only transmission but dispersal as well. For example, capsaicinoids in wild *Capsicum* pepper plants deter their most damaging seed pathogen (Tewksbury et al., 2008) and they also deter poor dispersal agents. Birds, in contrast, effectively disperse *Capsicum* seeds and they are not deterred by the maximal concentrations of capsaicin in the placental tissues surrounding the seeds since they are capsaicin-insensitive.

Dispersed seeds of wild plants must continue to benefit from strong defense, particularly when banked in soil for longer periods of time. Microbes themselves (i.e., primary symbionts) are known to contribute to banked seed defense (Chee-Sanford et al., 2006) and this mechanism is in addition to four other proposed mechanisms (Dalling et al., 2011) and a newly proposed, fifth, enzyme-based one (Fuerst et al., 2014). Still, up to 90% of all seeds die while in the seed bank (i.e., soil) (Cook, 1980). Fungal pathogens are behind much of that loss (Kirkpatrick and Bazzaz, 1979). In nature, most seedlings die in ways that could easily involve the primary symbiont or its absence. DSE fungi can be more quickly recruited by seedlings with primary symbionts than those without (Ridout et al., unpublished), and DSE fungi have mostly positive effects on plants (Mandyam and Jumpponen, 2005).

The importance to humanity of the seeds of cereal grasses (e.g., rice, maize, wheat) can be appreciated in the strong parallel between their domestication and the rise of human civilization (Diamond, 2002). Today, seeds of cereals provide almost half of all calories consumed by humans; other edible seeds (e.g., nuts, legumes, pseudocereals, spice seeds) provide additional calories (Kearney, 2010), vitamins, minerals, antioxidants (Halvorsen et al., 2006), and even probiotics in the form of seedborne microbes (Pathak, 2013). Ecologically, seeds and/or allied fruits are not only units of consumption but units of dispersal, succession, invasion, and survival of environmental adversity. Germination unleashes strong competition among seedlings that shapes wild communities and in crop situations demands management. Mortality takes most of its toll of individual plants among seedlings. Inasmuch as seeds are evolutionarily and ecologically essential to individual and community plant processes, attention to maternally transmitted microbes is overdue. Maternally transmitted microbes might even be the place to begin to look for a core microbiome in plants (Busby et al., 2017).

## Author Contributions

GN conceived of the 98-species study and wrote this Hypothesis and Theory paper. AH conducted the study. PB and MR contributed to the study and to the paper.

## Conflict of Interest Statement

The authors declare that the research was conducted in the absence of any commercial or financial relationships that could be construed as a potential conflict of interest.
